# Herbst–multibracket appliance treatment: is there an association between lower incisor position changes and the development of labial gingival recessions?

**DOI:** 10.1007/s00056-020-00272-0

**Published:** 2021-01-13

**Authors:** S. Südwasser, N. C. Bock, J. Jost, S. Killat, S. Ruf

**Affiliations:** 1 Department of Orthodontics, University of Giessen, Schlangenzahl 14, 35392 Giessen, Germany; 2 Private Practice, Limburg, Germany; 3 Private Practice, Stuttgart, Germany

**Keywords:** Malocclusion, Angle class II, Oral health, Periodontal disease, Labial gingival recessions, Inclination of lower incisors, Malokklusion Angle-Klasse II, Mundgesundheit, Parodontalerkrankungen, Labiale gingivale Rezessionen, Inklination unterer Inzisivi

## Abstract

**Purpose:**

To assess a potential association between lower incisor (LI) position changes during Herbst–multibracket appliance (Herbst–MBA) treatment and the development of labial gingival recessions (LGR).

**Methods:**

All class II patients (Department of Orthodontics, University of Giessen, Giessen, Germany) who had undergone Herbst–MBA treatment until 2015 with study models and lateral cephalograms available from before (T0) and after treatment plus ≥24 months of retention (T3) were included (*n* = 259). Lateral cephalograms were evaluated regarding LI position changes: iiL/ML (angle between LI long axis and mandibular plane [MP]), ii-ML_Pg_ (distance between LI incisal edge and a line perpendicular to MP through pogonion), apex-ML_Pg_ (distance between LI apex and a line perpendicular to MP through pogonion), ii-ML_ii_ (distance between LI incisal edge and MP on a line perpendicular to MP through incisal edge). Using study models the distance between the cementoenamel junction and the deepest point of the gingival margin was defined as LGR.

**Results:**

The following cephalometric mean changes were recorded (T0–T3): iiL/ML +5.9 ± 5.76° (*p* = 0.929), ii-ML_Pg_ −0.2 ± 0.25 mm (*p* = 0.430), apex-ML_Pg_ +0.1 ± 0.32 mm (*p* = 0.363), ii-ML_ii_ +0.1 ± 0.36 mm (*p* = 0.206). The mean increase of LGR magnitude measured on the study models was 0.1 ± 0.35 mm. However, no association with the cephalometric LI position changes was found (|R| ≤ 0.2).

**Conclusion:**

There is no association between the amount of LI position changes and the development of LGR during Herbst–MBA treatment plus retention. Nevertheless, individual predisposition or excessive treatment changes and extraordinary treatment approaches, respectively, might still lead to development of LGR.

## Abstract (de)

**Ziel:**

Ermittlung eines möglichen Zusammenhangs zwischen Positionsveränderungen der unteren Inzisiven (UI) während Herbst-Multibracket-Apparatur(Herbst-MBA)-Behandlung und der Entwicklung labialer gingivaler Rezessionen (LGR).

**Methoden:**

Eingeschlossen wurden alle Klasse-II-Patienten (Abteilung für Kieferorthopädie, Justus-Liebig-Universität Gießen), die bis 2015 eine Herbst-MBA-Therapie durchlaufen hatten und von denen Studienmodelle und Fernröntgenseitenbilder von vor (T0) und nach der Behandlung sowie ≥24 Monate Retentionsphase (T3) vorlagen (*n* = 259).

Fernröntgenseitenbilder wurden hinsichtlich der Positionsveränderungen der UI ausgewertet: iiL/ML (Winkel zwischen UI-Längsachse und Unterkieferebene [UE]), ii-ML_Pg_ (Abstand zwischen UI-Schneidekante und einer Linie senkrecht zu UE durch Pogonion), apex-ML_Pg_ (Abstand zwischen UI-Apex und einer Linie senkrecht zu UE durch Pogonion), ii-ML_ii_ (Abstand zwischen UI-Schneidekante und UE auf einer Linie senkrecht zu UE durch die Schneidekante). An Studienmodellen wurde der Abstand zwischen der Schmelz-Zement-Grenze und dem tiefsten Punkt des Zahnfleischrands als LGR gemessen.

**Ergebnisse:**

Die folgenden kephalometrischen Veränderungen traten auf (T0–T3): – iiL/ML +5,9 ± 5,76° (*p* = 0,929), – ii-ML_Pg_ −0,2 ± 0,25 mm (*p* = 0,430), – apex-ML_Pg_ +0,1 ± 0,32 mm (*p* = 0,363), – ii-ML_ii_ +0,1 ± 0,36 mm (*p* = 0,206): Die mittlere LGR-Veränderung – gemessen an Studienmodellen – betrug 0,1 ± 0,35 mm. Ein Zusammenhang mit den kephalometrischen Veränderungen der UI konnte nicht ermittelt werden (|R| ≤ 0,2).

**Schlussfolgerung:**

Es gibt keinen Zusammenhang zwischen dem Ausmaß der UI-Positionsveränderungen und der Entwicklung von LGR während einer Herbst-MBA-Behandlung einschließlich der Retentionsphase. Dennoch kann es durch individuelle Prädisposition oder exzessive therapeutische Veränderungen bzw. außergewöhnliche Behandlungsstrategien zur Entwicklung von LGR kommen.

## Introduction

Labial gingival recessions (LGR) result not only in esthetic impairment but are also associated with tooth hypersensitivity and a greater susceptibility to root caries [36].

Since the 1970s it has been hypothesized that the development of LGR is a result of bone dehiscences after proclination of lower incisors [5, 34]. However, it still remains controversial whether orthodontic proclination of the lower incisors really promotes the development of LGR [15, 18, 23]. Nevertheless, proclination of lower incisors has been described as a risk factor [1, 3, 11].

Protrusion and proclination of the lower incisors are side effects of Herbst treatment [12, 16, 17, 22, 24, 25, 27, 31]. Previous investigations have already assessed whether Herbst treatment and the respective proclination of lower incisors is associated with LGR [7–9, 26, 31]. The highest incidence was seen in lower incisors. However, due to the fairly small average magnitude of LGR development, the clinical relevance was considered insignificant. In another study using cone-beam computed tomography to investigate whether Herbst treatment induces alveolar bone loss around the mandibular incisors, no statistically significant effects were found regarding the vertical alveolar bone level or the alveolar bone thickness [32].

An investigation assessing lower incisor inclination and position changes during Herbst treatment (for an average of 6 months) in 98 patients revealed no direct relationship with LGR development, neither for patients with high (16.4 ± 1.9°) nor for patients with low inclination changes of the lower incisors (2.7 ± 1.7°) [31]. Also Renkema et al. [29, 30] found no association between lower incisor inclination changes during treatment and the development of LGR in multibracket appliance (MBA) patients over a period of 5 years postretention. Another study evaluated the long-term prevalence of gingival recessions focusing on the effects of mandibular incisor proclination. They concluded that orthodontic treatment is not a major risk factor for recession development [23]. In contrast, an investigation published in 2019 described an association between gingival recessions and incisor proclination (>10°) in 25% of 126 patients after 7.3 years of active fixed appliance treatment and retention. The authors also examined the relationship between the development of recessions and mandibular morphology; only the height of the symphysis was found to be related to the prevalence of lingual recessions on the lateral incisors [28].

Thus, there is currently only little and inconclusive data regarding an association between the amount of proclination of the lower incisors and the development of LGR during Herbst–MBA treatment plus retention—especially not long-term or for large cohorts. Most previous long-term investigations examined patients treated with MBA only [29], patients with large proclination values of the lower incisors after treatment [30] or analyzed small patient samples [26].

Therefore, the aim of the present investigation was to assess a large, representative sample of consecutive class II patients treated with a Herbst–MBA for inclination/position changes of the lower incisors and a possible association with the incidence or progression of LGR on the lower incisors.

## Materials and methods

After obtaining ethical approval (No. 80/14), the records of all class II patients who had been treated with a Herbst–MBA at the study center (Department of Orthodontics, University of Giessen, Giessen, Germany) since 1986 were screened for the following inclusion criteria:

- Active treatment completed by 1 January 2015
- Lateral cephalograms and unaltered study casts available from before treatment (T0) and after Herbst–MBA treatment plus ≥24 months of retention (T3); both records had to be taken at the same occasion (±0 months).

In addition, the lateral cephalograms from immediately after the Herbst period (T1) and after the subsequent MBA period (T2) were evaluated if available.

**Fig. 1:**
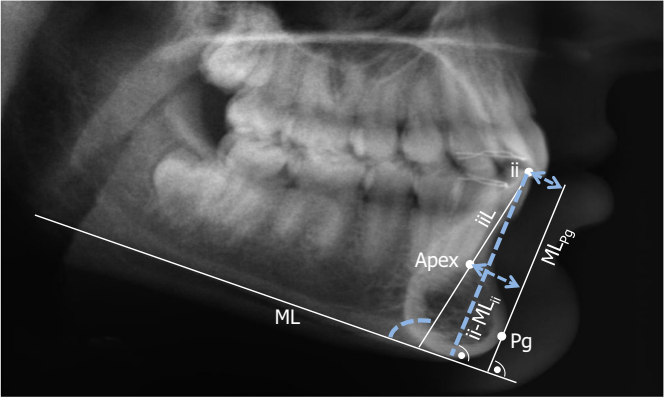
Cephalometric evaluation: The reference points and lines as well as the variables (*white*) used for the assessment of proclination and position of the lower incisors (LI, *blue*): iiL/ML—angle between the LI long axis and the mandibular plane; ii-ML_Pg_—distance between the LI incisal edge and a line perpendicular to the mandibular plane through pogonion; apex-ML_Pg_—distance between the LI apex and a line perpendicular to the mandibular plane through pogonion; ii-ML_ii_—distance between the LI incisal edge and mandibular plane on a line perpendicular to the mandibular plane through the incisal edge Kephalometrische Auswertung: Referenzpunkte und -linien (*weiß*) sowie die zur Messung der Proklination und Position der unteren Inzisiven (UI) verwendeten Variablen (*blau*): iiL/ML – Winkel zwischen UI-Längsachse und UE (Unterkieferebene); ii-ML_Pg_ – Abstand zwischen UI-Schneidekante und einer Senkrechten zu UE durch Pogonion; apex-ML_Pg_ – Abstand zwischen UI-Apex und einer Senkrechten zu UE durch Pogonion; ii-ML_ii_ – Abstand zwischen UI-Schneidekante und UE auf einer Linie senkrecht zu UE durch die Schneidekante

The lower incisor inclination/position was evaluated on lateral cephalograms using the following variables (Fig. 1):

- iiL/ML (angle between the lower incisors’ long axis and the mandibular plane)
- ii-ML_Pg_ (distance between the lower incisors’ incisal edge and a line perpendicular to the mandibular plane through pogonion)
- apex-ML_Pg_ (distance between the lower incisors’ apex and a line perpendicular to the mandibular plane through pogonion)
- ii-ML_ii_ (distance between the lower incisors’ incisal edge and the mandibular plane on a line perpendicular to the mandibular plane through the incisal edge).

The measurements were made to the nearest 0.5° or 0.5 mm. No correction was performed for radiographic enlargement (approximately 7% in the median plane). Inclination/position changes were calculated as the difference between the measurements undertaken at T0, T1, T2, and T3.

**Fig. 2:**
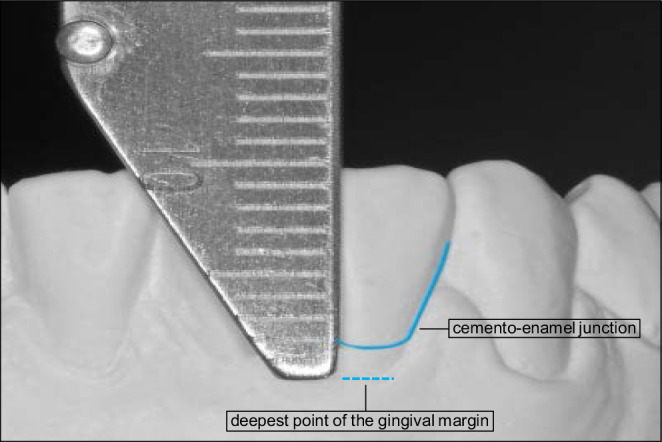
Labial gingival recessions (LGR) measurement on study casts. The *dashed line* marks the cementoenamel junction; the *solid line* marks the deepest point of the gingival margin. The distance between the two lines was measured to the nearest 0.5 mm using a manual caliper LGR(labiale gingivale Rezession)-Messung am Studienmodell. Die *gestrichelte Linie* markiert die Schmelz-Zement-Grenze, die *durchgezogene Linie* den tiefsten Punkt des Zahnfleischrandes. Der Abstand zwischen den beiden Linien wurde mittels manueller Schieblehre gemessen (Genauigkeit 0,5 mm)

The distance between the cementoenamel junction and the deepest point of the gingival margin was assessed and—in case of a positive value—defined as LGR (Fig. 2). Measurements were made to the nearest 0.5 mm using a manual caliper (HSL247-52, Karl Hammacher GmbH, Solingen, Germany).

One operator (J.J.) performed all measurements for class II:1 and one operator (S.K.) performed all measurements for class II:2 on study models as part of their doctoral thesis [7, 8]. To assess observer reliability, the study models of 20 randomly selected class II:1 and class II:2 patients each were evaluated twice after 4 weeks and the Kendall’s tau correlation coefficient was calculated. The respective values range between 0.71 for class II:1 and 0.84 for class II:2, indicating high consistency [10].

Due to severe gingival swelling/hyperplasia being often present upon debonding, the study casts from directly after debonding the Herbst appliance (T1) and the MBA (T2) were excluded and only those from after retention (T3), where marked swelling is unusual, were used. In any case, the inclusion of patients was performed irrespective of treatment outcome.

For the evaluation of the LGR changes under Herbst–MBA treatment plus retention, both the maximum change in a single lower incisor (most severe value per patient) and the mean of all four lower incisors were considered.

All cephalometric measurements were performed by one single operator blinded for the LGR data (S.S.). To assess the observer reliability, 20 randomly selected cephalograms were evaluated twice with an intermediate time interval of at least 4 weeks. The Pearson correlation coefficient was ≥0.86 for all variables, which corresponds to a very high consistency [10].

All statistical analyses were performed using the software IBM® SPSS® Statistics Version 25 (IBM Corporation, Armonk, NY, USA). For the changes which occurred during Herbst–MBA treatment and retention (T0–T3), an explorative statistical analysis was performed. The mean, standard deviation, minimum, maximum and median values are given for all variables. Kendall’s tau test was used for data analysis and to assess a possible interrelation between tooth-position changes and the incidence or progression of LGR. The level of significance was set at *p* *≤* 0.05.

## Results

A total of 259 class II patients (59.5% females, 40.5% males) with a pretreatment age of 14.6 ± 3.7 years (range 10.3–43.5 years) fulfilled all inclusion criteria and were included in the investigation.

The mean duration for active treatment (Herbst–MBA) was 23.3 ± 7.7 months (range 11.2–52.6 months); the mean total observation period (active treatment including retention) was 58.6 ± 11.5 months (range 36.1–212.7 months). For retention, upper and lower bonded retainers (canine to canine) were used in 141 patients (54.4%), in 11 patients (4.3%) a bonded retainer was used in the upper jaw only, while in 89 patients (34.4%) a bonded retainer was only used in the lower jaw. The remaining 6.9% had removable upper and/or lower retention plates or no retention at all.

Table 1 shows the inclination and position values of the lower incisors as well as the magnitude of LGR at all four observation time points. The corresponding changes are given in Table 2.

**Table 1:** Position and inclination values of the lower incisors determined from lateral cephalograms from before treatment (T0), after Herbst treatment (T1), after subsequent multibracket appliance (MBA) treatment (T2) and after at least 24 months of retention (T3) as well as labial gingival recession (LGR) magnitude values from before treatment (T0) and after active treatment plus at least 24 months of retention (T3). The mean value and standard deviation as well as the median, minimum and maximum values are given Positions- und Inklinationswerte der unteren Schneidezähne ermittelt aus den Fernröntgenseitenbildern der Zeitpunkte vor Behandlung (T0), nach Herbst-Behandlung (T1), nach anschließender MBA(Multibracket-Apparatur)-Behandlung (T2), nach mindestens 24 Monaten Retention (T3) sowie das Ausmaß von LGR (labiale gingivale Rezession) der Zeitpunkte vor Behandlung (T0) und nach aktiver Behandlung plus mindestens 24 Monaten Retention (T3). Mittelwert und Standardabweichung sowie Median, Minimum und Maximum sind angegeben

			T0 (*n* = 259)					T1 (*n* = 249)					T2 (*n* = 249)					T3 (*n* = 259)				
			*Mean*	*SD*	*Min*	*Max*	*Median*	*Mean*	*SD*	*Min*	*Max*	*Median*	*Mean*	*SD*	*Min*	*Max*	*Median*	*Mean*	*SD*	*Min*	*Max*	*Median*
*Lateral cephalometric evaluation*	*iiL/ML*	°	97.6	7.25	69.5	112.0	98.0	109.9	8.32	82.0	128.0	110.0	104.0	7.40	79.0	126.5	104.0	103.5	7.52	77.0	129.0	103.5
	*ii-ML*_*Pg*_	mm	0.1	0.38	0.1	2.6	0.9	0.6	0.38	−0.3	2.2	0.5	0.8	0.38	−0.1	2.5	0.8	0.8	0.39	0.0	2.5	0.8
	*Apex-ML*_*Pg*_	mm	1.3	0.22	0.2	2.0	1.3	1.4	0.22	0.7	2.0	1.4	1.4	0.22	0.8	2.0	1.4	1.4	0.24	0.1	2.0	1.4
	*ii-ML on ML*_*ii*_	mm	4.1	0.42	1.1	5.2	4.2	4.0	0.37	3.1	5.2	4.0	4.1	0.38	3.3	5.2	4.1	4.2	0.38	3.3	5.2	4.2
*Study model evaluation*	*Tooth 32*	mm	0.0	0.13	0.0	1.0	0.0	n.a.					n.a.					0.1	0.30	0.0	2.0	0.0
	*Tooth 31*	mm	0.1	0.24	0.0	2.0	0.0	n.a.					n.a.					0.1	0.37	0.0	2.5	0.0
	*Tooth 41*	mm	0.1	0.35	0.0	4.0	0.0	n.a.					n.a.					0.2	0.51	0.0	4.0	0.0
	*Tooth 42*	mm	0.0	0.27	0.0	4.0	0.0	n.a.					n.a.					0.2	0.58	0.0	4.0	0.0
	*Average* (Teeth 32–42)	mm	0.0	0.18	0.0	1.5	0.0	n.a.					n.a.					0.1	0.31	0.0	2.1	0.0

*n.a.* not available, *SD* standard deviation, *Min* minimum, *Max* maximum

**Table 2:** Position and inclination changes of the lower incisors during Herbst treatment (T0–T1), during subsequent multibracket appliance (MBA) treatment (T1–T2), during retention (T2–T3) and the total observation period (T0–T3) as well as labial gingival recession (LGR) magnitude changes during treatment plus retention (T0–T3). The mean value and standard deviation as well as the median, minimum and maximum values are given Positions- und Inklinationsveränderungen der unteren Schneidezähne während Herbst-Behandlung (T0–T1), anschließender MBA(Multibracket-Apparatur)-Behandlung (T1–T2), Retention (T2–T3) und gesamter Beobachtungsperiode (T0–T3), sowie Ausmaßveränderungen der LGR (labiale gingivale Rezession) während aktiver Behandlung plus Retention (T0–T3). Mittelwert und Standardabweichung sowie Median, Minimum und Maximum sind angegeben

			T0–T3 (*n* = 259)					T0–T1 (*n* = 249)					T1–T2 (*n* = 249)					T2–T3 (*n* = 249)				
			Mean	SD	Min	Max	Median	Mean	SD	Min	Max	Median	Mean	SD	Min	Max	Median	Mean	SD	Min	Max	Median
*Lateral cephalometric evaluation*	*iiL/ML*	°	5.9	5.76	−17.5	31.5	6.0	12.3	6.20	−5.0	41.5	12.0	−5.9	5.80	−25.0	12.5	−6.0	−0.5	3.80	−16.0	9.5	−0.5
	*ii-ML*_*Pg*_	mm	−0.2	0.25	−0.9	1.3	−0.2	−0.4	0.17	−1.0	0.0	−0.4	0.2	0.20	−0.6	1.3	0.2	0.0	0.15	0.2	1.1	0.0
	*Apex-ML*_*Pg*_	mm	0.1	0.32	−0.6	4.5	0.1	0.1	0.17	−0.7	1.0	0.1	0.0	0.30	−0.5	0.6	0.0	0.0	0.19	−0.9	1.3	0.0
	*ii-ML on ML*_*ii*_	mm	0.1	0.36	−0.7	3.3	0.1	−0.1	0.35	−1.0	3.3	−0.1	0.1	0.16	−0.4	0.6	0.1	0.1	0.14	−0.4	1.0	0.1
*Study model evaluation*	*Tooth 32*	mm	0.1	0.30	1.0	2.0	0.0	n.a.					n.a.					n.a.				
	*Tooth 31*	mm	0.1	0.45	−2.0	2.5	0.0	n.a.					n.a.					n.a.				
	*Tooth 41*	mm	0.1	0.52	−2.0	4.0	0.0	n.a.					n.a.					n.a.				
	*Tooth 42*	mm	0.1	0.53	−1.0	4.0	0.0	n.a.					n.a.					n.a.				
	*Average* (Teeth 32–42)	mm	0.1	0.35	−1.5	2.2	0.0	n.a.					n.a.					n.a.				

*n.a.* not available, *SD* standard deviation, *Min* minimum, *Max* maximum

### Lateral cephalometric evaluation

**Fig. 3:**
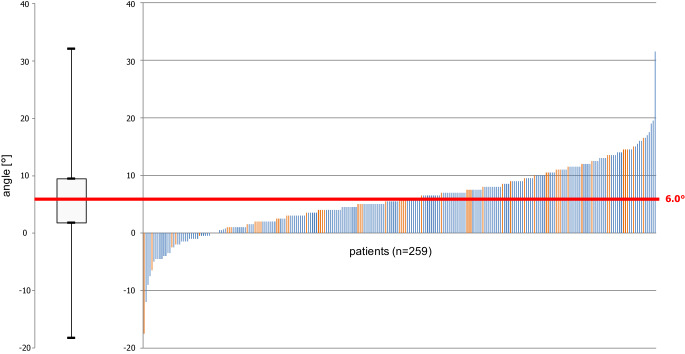
Lower incisor inclination/angulation changes (iiL/ML) during Herbst–multibracket appliance (MBA) treatment plus at least 24 months of retention (T0–T3) in all 259 patients. Patients who developed at least one labial gingival recessions (LGR) (*n* = 54) are marked in *orange* Inklinations‑/Angulationsveränderungen (iiL/ML) der unteren Schneidezähne aller 259 Patienten während Herbst-MBA(Multibracket-Apparatur)-Behandlung plus mindestens 24 Monaten Retention (T0–T3). Patienten, die mindestens eine LGR (labiale gingivale Rezession) entwickelt haben (*n* = 54), sind *orange* markiert

While an increase of the lower incisors’ inclination (iiL/ML) by 12.3 ± 6.20° had occurred during the Herbst period (T0–T1), this value decreased by 5.9 ± 5.80° during the subsequent MBA period and by a further 0.5 ± 3.80° during retention (T2–T3). Thus, the resulting overall change (T0–T3) was an increase by 5.9 ± 5.76° (*p* = 0.929; Fig. 3, Table 2). However, a large interindividual range from −17.5° to 31.5° was seen.

The distance between the lower incisors’ incisal edge and a line perpendicular to the mandibular plane through pogonion (ii-ML_Pg_) decreased by 0.4 ± 0.17 mm during Herbst treatment (T0–T1), increased during MBA treatment by 0.2 ± 0.20 mm and remained unchanged during retention (0.0 ± 0.15 mm). Thus, for the overall observation period (T0–T3) a decrease by 0.2 ± 0.25 mm (*p* = 0.430) occurred.

The distance between the lower incisors’ apex and a line perpendicular to the mandibular plane through pogonion (apex-ML_Pg_) increased slightly by 0.1 ± 0.32 mm (*p* = 0.363) during Herbst treatment (T0–T1). It remained stable both during the MBA period (0.0 ± 0.30 mm; T1–T2) and retention (0.0 ± 0.19 mm; T2–T3). In total, an increase by 0.1 ± 0.32 was seen during the overall period (T0–T3).

The distance between the lower incisors’ incisal edge and the mandibular plane on a line perpendicular to the mandibular plane through the incisal edge (ii-ML_ii_) decreased slightly during the Herbst period (0.1 ± 0.35 mm) and increased by 0.1 mm both during MBA treatment and the subsequent retention period (T1–T2: 0.1 ± 0.16 mm; T2–T3: 0.1 ± 0.14 mm). Thus, the resulting overall change was an increase by 0.1 ± 0.36 mm (*p* = 0.206) during the total observation period (T0–T3).

### Study model evaluation

The average LGR magnitude was 0.0 ± 0.18 mm prior to treatment (T0) and 0.1 ± 0.31 mm after treatment and retention (T3; Table 1). Thus, the respective LGR increase was 0.1 ± 0.35 mm (T0–T3; Table 2). The lowest increase was seen for both lower lateral incisors: 90% of the patients showed no LGR development on teeth 32 and 42. On tooth 31, no LGR increase was seen in 84% of patients, while on tooth 41 no LGR increase was observed in 86% of patients.

**Fig. 4:**
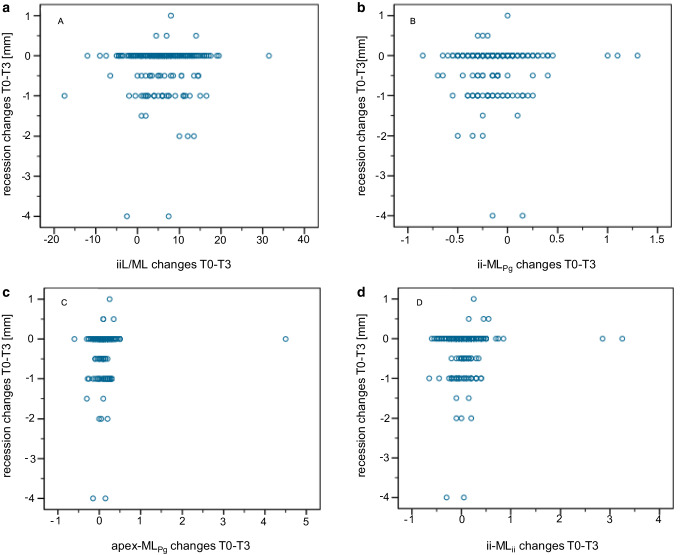
Distribution of the lower incisors’ position changes (T0–T3) relative to the respective labial gingival recessions (LGR) changes. For each of the four cephalometric variables (**a** iiL/ML, **b** ii-ML_Pg_, **c** Apex-ML_Pg_, **d** ii-ML_ii_) the possibility of an association with LGR development is shown (|R| values: iiL/ML = 0.055, ii-ML_Pg_ = −0.059, apex-ML_Pg_ = −0.061, ii-ML_ii_ = −0.067) Verteilung der Positionsveränderungen der unteren Schneidezähne (T0–T3) relativ zu den entsprechenden LGR(labiale gingivale Rezession)-Veränderungen. Für jede der 4 kephalometrischen Variablen (**a** iiL/ML, **b** ii-ML_Pg_, **c** Apex-ML_Pg_, **d** ii-ML_ii_) ist die Möglichkeit für einen Zusammenhang mit der Entstehung/Progredienz von LGR dargestellt (|R|-Werte: iiL/ML = 0,055, ii-ML_Pg_ = −0,059, apex-ML_Pg_ = −0,061, ii-ML_ii_ = −0,067)

No correlation between the lower incisors’ position changes and the incidence or progression of LGR during Herbst–MBA treatment including retention (T0–T3) could be determined (Kendall tau b, |R| ≤ 0.2; Fig. 4).

## Discussion

In addition to the esthetic impairment of lower incisors, recessions might cause tooth hypersensitivity and greater susceptibility for root caries [36]. Therefore, the aim of the present investigation was to assess a possible association between the development of LGR and lower incisor (32-42) inclination and position changes during class II Herbst–MBA treatment and a subsequent retention period of ≥24 months in a large, representative sample of consecutive patients.

The morphologic LGR data determined from study casts have been previously published for II:1 [8] and II:2 [7] malocclusions; the cephalometric proclination values and the possible respective association were previously reported in an oral presentation at the 95th Congress of the European Orthodontic Society in 2019 and were published as part of the respective abstract [35].

### Materials and methods

All class II patients who underwent Herbst–MBA treatment at the study center with available lateral cephalograms and study casts from before and after treatment plus ≥24 months of retention were included. The final patient sample (*n* = 259) is large compared with previous articles on the same topic. The patient sample was homogenous in terms of class II malocclusion and the appliances used (Herbst and MBA). However, treatment had been accomplished by several practitioners including postgraduate students, which might have had a minor impact on treatment outcome [21] and treatment duration [19].

In addition, the retention regime was not uniform as the patient sample was retrospectively collected and treated over a period of almost 30 years. During the early years of Herbst–MBA treatment, the standard retention protocol comprised of mainly removable appliances (predominantly Hawley retainers), while fixed retention was established during the later years. In any case, the inclusion of patients was performed irrespective of treatment outcome.

### Results

The overall increase in lower incisor inclination during Herbst–MBA treatment plus retention was 5.9 ± 5.76°. It is difficult to compare this value with previous results from the literature. Only one publication [31] contains data on almost the same treatment protocol (Herbst plus at least partially MBA). One study investigated the proclination changes of lower incisors (*n* = 126 patients) solely after MBA treatment and retention [28]; the respective proclination values were lower than the present ones (2.7° for patients without LGR development, 3.0° for patients who developed at least one LGR). This might be due to the difference in treatment protocol.

Due to the large interindividual range of the amount of proclination during T0–T3, we additionally evaluated the inclination and position of the lower incisors separately for the Herbst phase and the MBA phase. While an average increase of 12.3 ± 6.20° had occurred during Herbst treatment, this value decreased by 5.9 ± 5.8° during MBA treatment and by further 0.5 ± 3.8° during retention. The amount of proclination during Herbst treatment is in concordance with previous studies (8.9–12.7°) [9, 14, 31, 38, 39]. The same holds true for the amount of reversion during subsequent MBA treatment [9, 27].

The present results showed no association between LGR development and the amount of proclination. As teeth with a minor degree of proclination were affected similarly by LGR as those with distinct proclination, respective values seem not to be directly attributable to the formation of LGR. This result is in concordance with the findings of other investigations and reviews in the literature [4, 13, 23, 26, 30, 31, 37]. Several studies found both proclined and nonproclined teeth to exhibit similar LGR development [13, 30, 31]. In contrast, other articles in the literature describe that orthodontic tooth movement might increase the risk for LGR development during retention [6, 33]. So, how can this controversy be explained? Obviously, the development of LGR is highly multifactorial [4, 28] with numerous factors (e.g., individual susceptibility, patients’ oral hygiene compliance and or technique, periodontal/symphysal morphology, gingival phenotype, bone wall thickness or bone fenestration) modifying clinical response. Therefore, LGR development of course still might occur during or after Herbst–MBA treatment due to individual predisposition, excessive general treatment changes or extraordinary treatment approaches, especially long-term. In line with the latter assumptions, Melsen and Allais demonstrated that the only important factors for the development of LGR were related to gingival morphology and periodontal health while the amount of incisor proclination was not correlated to LGR development [20]. Furthermore, aggressive tooth brushing might be an important etiologic factor [2].

### Limitations

The retrospective study design (which includes the fact that, for example, no data on oral hygiene during treatment were assessed) certainly has to be considered as a limitation. In addition, the growth pattern was not considered and no comparison to an untreated control group was made, as no respective data are available and it would not be justifiable to generate respective data for ethical reasons. Furthermore, for the reasons mentioned above, we were unable to evaluate the study models from directly after active treatment (Herbst–MBA), making it impossible to say whether the respective LGRs occurred during active treatment or retention. Finally, we did not investigate a possible development of gingival recessions on the opposite, lingual side, which could be done in further research as well as the consideration of intraoral photographs.

## Conclusion

The data of the present investigation and their comparison with the literature show that Herbst–MBA treatment cannot be considered a clinically relevant risk factor for LGR development on the lower incisors. Nevertheless, individual predisposition or excessive treatment changes or extraordinary treatment approaches might still lead to LGR development.
